# Impact of adequate lymph nodes dissection on survival in patients with stage I rectal cancer

**DOI:** 10.3389/fonc.2022.985324

**Published:** 2022-11-18

**Authors:** Peng-Lin Liu, Dan-Dan Wang, Cheng-Jian Pang, Li-Ze Zhang

**Affiliations:** Department of Anorectum, The Affiliated Hospital of Qingdao University, Shandong, China

**Keywords:** rectal cancer, proctectomy, lymph nodes dissection, cancer-specific survival, SEER

## Abstract

**Background and Aims:**

The NCCN guidelines recommended an assessment of ≥ 12 lymph nodes (LN) as an adequate LN dissection (LND) for rectal cancer (RC). However, the impact of adequate LND on survival in stage I RC patients remained unclear. Thus, we aimed to compare the survival between stage I RC patients with adequate and inadequate LND.

**Methods:**

A total of 1,778 stage I RC patients in the SEER database from 2010 to 2017 treated with radical proctectomy were identified. The association between ≥ 12 LND and survival was examined using the multivariate Cox regression and the multivariate competing risk model referenced to < 12 LND.

**Results:**

Stage I RC patients with ≥ 12 LND experienced a significantly lower hazard of cancer-specific death compared with those with < 12 LND in both multivariate Cox regression model (adjusted HR [hazard ratio], 0.44, 95% CI, 0.29-0.66; *P* < 0.001) and the multivariate competing risk model (adjusted subdistribution HR [SHR], 0.45, 95% CI, 0.30-0.69; *P* < 0.001). Further, subgroup analyses performed by pT stage. No positive association between ≥ 12 LND and survival was found in pT1N0 RC patients (adjusted HR: 0.62, 95%CI, 0.32-1.19; *P* = 0.149; adjusted SHR: 0.63, 95%CI, 0.33-1.20; *P* = 0.158), whereas a positive association between ≥ 12 LND and survival was found in pT2N0 RC patients (adjusted HR: 0.35, 95%CI, 0.21-0.58; *P* < 0.001; adjusted SHR: 0.36, 95%CI, 0.21-0.62; *P* < 0.001).

**Conclusions:**

The long-term survival benefit of adequate LND was not found in pT1N0 but in pT2N0 RC patients, which suggested that pT2N0 RC patients should be treated with adequate LND and those with inadequate LND might need additional therapy.

## Introduction

Colorectal cancer is the third most frequently diagnosed cancer and the second leading cause of cancer death worldwide. Of these, an estimated 732,210 cases of rectal cancer (RC) will occur, and an estimated 339,022 people will die of RC in 2020 (1). Traditionally, the radical curative treatment for RC has been proctectomy, which involved lymph nodes (LN) dissection (LND). An assessment of a minimum of 12 LND (≥ 12 LND) is recommended in NCCN Guidelines Version 2.2022. The adequate LND will reduce the risk of metastatic LN residual, and then optimize locoregional control and tumor staging. For example, the node-negative pT1/pT2 RC patients with few LND might not be truly node-negative but rather understaging, the pN0 could be staged pN1, even pN2 with more LND (2–5). Moreover, the isolated tumor cells and micrometastasis in LN are considered the risk factors that could increase the rate of local recurrence and decrease the long-term survival of RC patients (6–8). Whereas local excision, which typically did not involve LND, was increasingly used in the treatment of stage I RC patients which helped to preserve the anus and reduce the morbidity and mortality resulting from radical proctectomy and further enhance the quality of life (9). According to the American Society of Colon and Rectal Surgeons, the criteria for local excision included pT1 stage, well-to-moderately differentiated, less than 3 cm diameter, less than one-third of the bowel lumen circumference, and the absence of lymphovascular or perineural invasion (10, 11). Local excision was increasingly used for the treatment of pT1N0 RC patients, and previous studies have confirmed the compare oncological long-term survival between these patients treated with local excision and radical proctectomy (9, 12). However, it was surprising that local excision was also increasingly used for the treatment of pT2N0 RC patients who did not meet the criteria of local excision (9, 12). Thus, the survival benefit of adequate LND should be questioned for the treatment of stage I RC patients. In addition, stage II RC patients (i.e., pT3N0M0) with < 12 LND are thought to place patients at higher risk, and an additional adjuvant therapy might be taken into consideration for these patients (13). However, the association between adequate LND and survival in pT1/pT2N0 RC patients remained unclear, and no additional therapy was recommended for these patients.

Therefore, in the present study, we identified stage I RC patients treated with radical proctectomy in the Surveillance, Epidemiology, and End Results (SEER) database and further evaluated the association between adequate LND (≥ 12) and survival with inadequate LND (< 12) as a reference, separately for pT1N0 RC patients and pT2N0 RC patients.

## Materials and methods

### Study design

This was a retrospective cohort study of patients in the SEER database from 2010 to 2017 with stage I RC treated with radical proctectomy. Informed consent or institutional review was not required for the analyses of patients collected because the SEER database is publicly available.

### Patients

**Figure 1:**
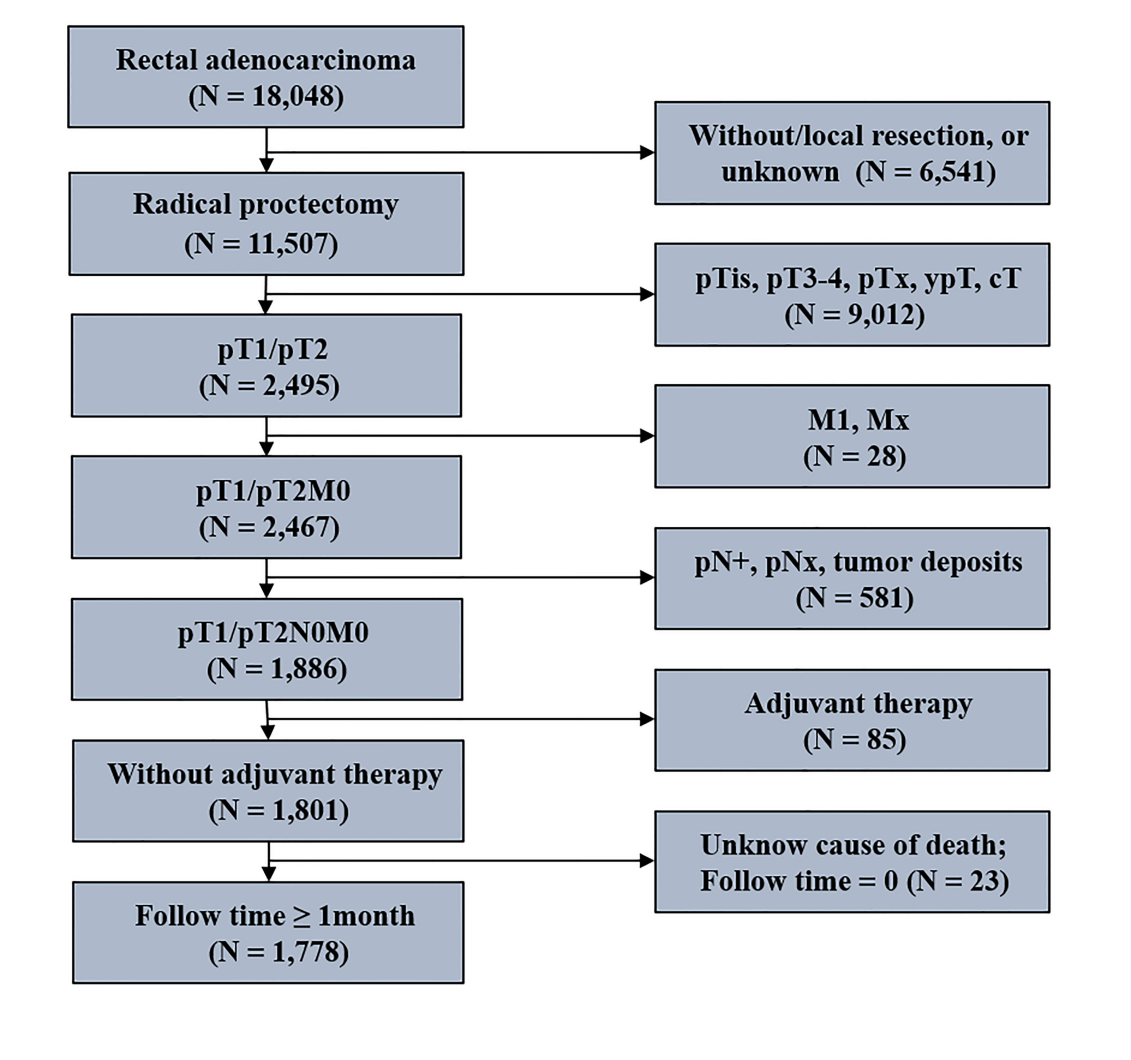
The flowchart of stage I rectal cancer patients included the process.

Patients’ data were collected from the SEER database using the National Cancer Institute’s SEER*Stat software (version 8.3.5; www.seer.cancer.gov). The detailed inclusion and exclusion criteria for stage I RC patients are shown in **Figure 1**. Patients were enrolled in 1) they were 18 years or older, 2) the histological type included adenocarcinoma, and mucinous adenocarcinoma, 3) they underwent radical proctectomy, 4) they had pT1/pT2N0M0 tumor, 5) they received no adjuvant therapy, 6) and they were actively followed up (follow-up time ≥ 1 month; known cause of death). Ultimately, 1,778 stage I RC patients with radical proctectomy were identified in the present study. The median follow-up time was 70 months, ranging between 46 and 94.

### Variables and outcomes

Stage I RC Patients were classified into inadequate LND (<12) and adequate LND (≥ 12) groups according to the number of LN examined. The assessment of 12 LN was chosen as the landmark of adequate LND following the NCCN guidelines (13). Patients’ demographic variables were age at diagnosis in 10-year increments, gender, and race (White, Black, and others). Tumor variables were pT stage (pT1 and pT2 stage), tumor grade (well/moderately differentiated and poor/anaplastic), tumor size (≤ 3 cm, > 3 cm, and unknown), CEA level (negative/unknown and positive) and perineural invasion (negative/unknown and positive).

### Statistical methods

Differences in patients’ demographics and tumor characteristics between inadequate LND and adequate LND were tested using the chi-square (χ^2^) test or Fisher’s exact test. The cancer-specific survival (CSS) was defined as RC’s time from diagnosis to death. For the competing risk model, death was classified into two groups: death related to RC and death not related to RC, which was considered a competing risk event. Patients who were still alive were censored at the date of the last contact. The CSS probabilities were calculated using the Kaplan-Meier method, and CSS probabilities differences between the groups were tested by the log-rank test. The multivariate Cox proportional hazards regression was performed to calculate the adjusted hazard ratio (HR) with 95% CI and evaluate the independent predictors of CSS. Taking into consideration the death not related to the RC, the competing risk model was performed to calculate the cumulative incidence of cancer-specific death (CSD) and the competing risk events, and the cumulative incidence differences between the groups were tested by the Gray’s test. The multivariate Fine and Gray’s competing risk regression model was performed to calculate the adjusted subdistribution HR (SHR) and 95% CI and evaluate the independent predictors of CSD (14). All statistical analyses were carried out using R statistical software (version 4.0.2, www.r-project.org). Two-sided P <.05 were considered statistically significant.

## Results

### Patients’ demographics and tumor characteristics

As shown in **Figure 1**, 1,778 stage I RC patients with radical proctectomy were identified in the present study. Of these, 425 patients with < 12 LND, and 1,353 patients with ≥ 12 LND. **Table 1** shows the differences in clinical characteristics between stage I RC patients with < 12 and ≥ 12 LND. Results showed that stage I RC patients with < 12 LND were diagnosed at an older age, with an earlier pT stage and a smaller size tumor compared with those with ≥ 12 LND. The differences in clinicopathological characteristics between patients with < 12 and ≥ 12 LND, separately for pT1N0 and pT2N0 RC patients, were summarized in **Supplemental Table 1**, **2**. Similar results were found that pT1N0 or pT2N0 RC patients with < 12 LND were diagnosed at an older age and with a smaller size tumor compared with those with ≥ 12 LND.

**Table 1:** Clinicopathological differences between stage I rectal adenocarcinoma patients with < 12 and ≥ 12 lymph nodes dissection.

Characteristic	TotalN = 1,778	< 12 lymph nodesN = 425	≥ 12 lymph nodesN = 1,353	*P-value*
Age of diagnosis (years)				0.006
< 50y	196 (6.0)	32 (7.5)	164 (12.1)	
< 60y	577 (32.5)	125 (29.4)	452 (33.4)	
< 70y	505 (28.4)	131 (30.8)	374 (27.6)	
≥ 70y	500 (28.1)	137 (32.2)	363 (26.8)	
Gender				0.065
Female	778 (43.8)	169 (39.8)	609 (45.0)	
Male	1,000 (56.2)	256 (60.2)	744 (55.0)	
Race				0.425
White	1,538 (86.5)	375 (88.2)	1,163 (86.0)	
Black	60 (3.4)	14 (3.3)	46 (3.4)	
Others	180 (10.1)	36 (8.5)	144 (10.6)	
pT stage				< 0.001
pT1	941 (52.9)	281 (66.1)	660 (48.8)	
pT2	837 (47.1)	144 (33.9)	693 (51.2)	
Tumor grade				0.292
Well/Moderately	1,544 (86.8)	373 (87.8)	1,171 (86.5)	
Poor/Anaplastic	142 (8.0)	27 (6.4)	115 (8.5)	
Unknown	92 (5.2)	25 (5.9)	67 (5.0)	
Tumor size				< 0.001
≤ 3cm	1,007 (56.6)	255 (60.0)	752 (55.6)	
> 3 cm	542 (30.5)	87 (20.5)	455 (33.6)	
Unknown	229 (12.9)	83 (19.5)	146 (10.8)	
CEA level				0.804
Negative/Unknown	1,620 (91.1)	389 (91.5)	1,231 (91.0)	
Positive	158 (8.9)	36 (8.5)	122 (9.0)	
Perineural invasion				0.663
Negative/Unknown	1,742 (98.0)	418 (98.4)	1,324 (97.9)	
Positive	36 (2.0)	7 (1.6)	29 (2.1)	

Values are n (%) unless otherwise defined.

### The association between lymph nodes dissection and prognosis

**Figure 2:**
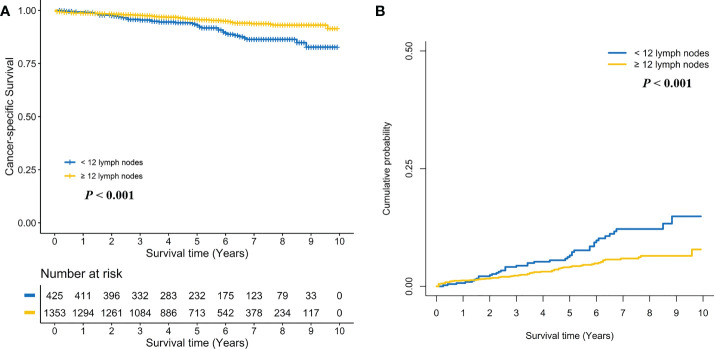
Comparison of cancer-specific survival **(A)** and cumulative probability of cancer-specific death **(B)** in stage I rectal cancer patients between < 12 and ≥ 12 lymph nodes dissection.

Kaplan-Meier survival curves are shown in **Figure 2A**. The 5-year CSS rate was 93.0% (95% CI, 90.4%–95.7%) for stage I RC patients with < 12 LND, and 95.7% (95% CI, 94.5%–96.9%) for stage I RC patients with ≥ 12 LND. The log-rank test showed that stage I RC patients with ≥ 12 LND had significantly better CSS rates compared with those with < 12 LND (P < 0.001, **Figure 2A**). To adjust for potential confounding factors, a multivariate Cox regression model was performed. stage I RC patients with ≥ 12 LND experienced a significantly lower hazard of CSD compared with those with < 12 LND (adjusted HR, 0.44, 95% CI, 0.29–0.66; *P* < 0.001, **Table 2**).

**Table 2:** The predictors of survival for pT1/pT2N0 rectal cancer patients in both multivariate Cox regression model and the multivariate competing risk model.

	Cox regression model		Competing risk model	
Characteristic	adjusted HR (95% CI)	*P-value*	adjusted SHR (95% CI)	*P-value*
Age of diagnosis				
< 50y	Reference		Reference	
< 60y	0.83 (0.34, 2.01)	0.671	0.82 (0.33, 2.04)	0.673
< 70y	1.62 (0.70, 3.74)	0.259	1.61 (0.68, 3.84)	0.280
≥ 70y	2.64 (1.18, 5.88)	0.018	2.40 (1.06, 5.44)	0.036
Gender				
Female	Reference		Reference	
Male	0.99 (0.66, 1.47)	0.944	0.97 (0.64, 1.46)	0.885
Race				
White	Reference		Reference	
Black	2.25 (0.90, 5.64)	0.082	2.31 (0.95, 5.63)	0.066
Others	0.84 (0.41, 1.74)	0.643	0.87 (0.42, 1.78)	0.698
pT stage				
pT1	Reference		Reference	
pT2	1.85 (1.18, 2.91)	0.007	1.83 (1.15, 2.92)	0.011
Tumor grade				
Well/Moderately	Reference		Reference	
Poor/Anaplastic	1.37 (0.73, 2.57)	0.325	1.37 (0.72, 2.61)	0.330
Unknown	0.24 (0.03, 1.79)	0.166	0.24 (0.03, 1.88)	0.175
Tumor size				
≤ 3cm	Reference		Reference	
> 3 cm	0.89 (0.57, 1.39)	0.609	0.88 (0.56, 1.39)	0.586
Unknown	0.64 (0.28, 1.42)	0.271	0.63 (0.27, 1.44)	0.270
CEA level				
Negative/Unknown	Reference		Reference	
Positive	1.90 (1.13, 3.17)	0.015	1.85 (1.10, 3.11)	0.020
Perineural invasion				
Negative/Unknown	Reference		Reference	
Positive	0.49 (0.07, 3.58)	0.485	0.48 (0.07, 3.27)	0.456
Lymph node dissection				
< 12	Reference		Reference	
≥ 12	0.44 (0.29, 0.66)	< 0.001	0.45 (0.30, 0.69)	< 0.001

Taking into consideration death not related to RC, the competing risk model was performed. The 5-year cumulative incidence of CSD rate was 6.6% (95% CI, 4.1%-9.1%) for stage I RC patients with < 12 LND, and 4.2% (95% CI, 3.0%-5.3%) for stage I RC patients with ≥ 12 LND. The Gray’s test showed that stage I RC patients with ≥ 12 LND had significantly lower CSD rates compared with those with < 12 LND (P < 0.001, **Figure 2B**). Also, a multivariate Fine and Gray’s competing risk regression model was performed to adjust for potential confounding factors. Stage I RC patients with ≥ 12 LND experienced a significantly lower hazard of CSD compared with those with < 12 LND (adjusted SHR, 0.45, 95% CI, 0.30-0.69; *P* < 0.001, **Table 2**).

**Figure 3:**
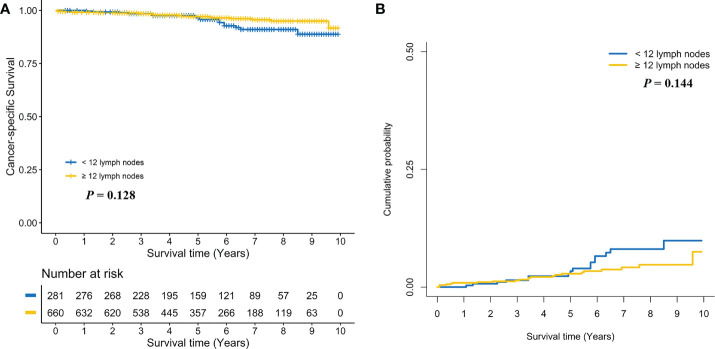
Comparison of cancer-specific survival **(A)** and cumulative probability of cancer-specific death **(B)** in pT1N0 rectal cancer patients between < 12 and ≥ 12 lymph nodes dissection.

**Figure 4:**
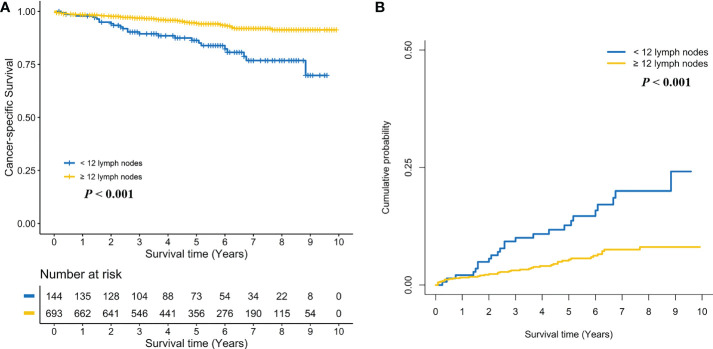
Comparison of cancer-specific survival **(A)** and cumulative probability of cancer-specific death **(B)** in pT2N0 rectal cancer patients between < 12 and ≥ 12 lymph nodes dissection.

Subgroup analyses were performed by pT stage. For pT1N0 RC patients, no positive association between ≥ 12 LND and CSS was found in the log-rank test (Five-year CSS: 97.1% *vs*. 96.4%, *P* = 0.128, **Figure 3A**) and the multivariate Cox regression model (adjusted HR: 0.62, 95%CI, 0.32-1.19; *P* = 0.149, **Supplemental Table 3**). Taking into consideration death not related to RC, no positive association between ≥ 12 LND and CSD was found in the Gray’s test (Five-year CSD: 2.8% *vs*. 3.4%, *P* = 0.144, **Figure 3B**) and the multivariate Fine and Gray’s competing risk regression model (adjusted SHR: 0.63, 95%CI, 0.33-1.20; P = 0.158, **Supplemental Table 3**). Conversely, a statistically significant survival benefit of ≥ 12 LND was found in pT2N0 RC patients in the log-rank test (Five-year CSS: 94.3% *vs*. 86.3%; *P* < 0.001, **Figure 4A**) and the multivariate Cox regression model (adjusted HR: 0.35, 95%CI, 0.21-0.58; *P* < 0.001, **Supplemental Table 4**). Also, taking into consideration death not related to RC, the statistically significant survival benefit of ≥ 12 LND did not change in pT2N0 RC patients in the univariate (Five-year CSD: 5.4% *vs*. 12.7%; *P* < 0.001, **Figure 4B**) and the multivariate competing risk model (adjusted SHR: 0.36, 95%CI, 0.21-0.62; *P* < 0.001, **Supplemental Table 4**).

Additionally, we conducted sensitivity analyses to assess the robustness of our results by excluding patients whose follow-up time was ≤ 3 months to account for bias due to surgery-associated death (15). The ≤ 3 months mortality for stage I RC patients with < 12 LND was 1.38% (6/436), which was insignificantly lower compared with 2.11% (28/1329) for stage I RC patients with ≥ 12 LND (*P* = 0.456). Landmark survival analyses were performed, and the statistically significant survival benefit of ≥ 12 LND were found in the stage I RC patients in the log-rank test (Five-year CSS: 96.3% *vs*. 93.2%, *P* < 0.001, **Supplemental Figure 1A**) and the multivariate Cox regression model (adjusted HR, 0.39; 95% CI, 0.25-0.59; *P* < 0.001, **Supplemental Table 5**) with exposure starting at > 3 months. Taking into consideration death not related to RC, the statistically significant survival benefit of ≥ 12 LND was no change in the stage I RC patients in the Gray’s test (Five-year CSD: 3.5% *vs*. 6.4%, *P* < 0.001, **Supplemental Figure 1B**) and the multivariate Fine and Gray’s competing risk regression model (adjusted SHR, 0.39; 95% CI, 0.25-0.61; *P* < 0.001, **Supplemental Table 5**). We also performed the same sensitivity analyses for stage I RC patients by pT stage. No positive association between ≥ 12 LND and survival was found in pT1N0 RC patients (**Supplemental Figures 2A, B** **;** **Supplemental Table 5**), whereas a positive association between ≥ 12 LND and survival was found in pT2N0 RC patients (**Supplemental Figures 3A, B** **;** **Supplemental Table 5**).

## Discussion

Traditionally, proctectomy along with adequate LND is the standard of surgical treatment for the vast majority of RC patients. The adequate LND will reduce the risk of metastatic LN residual and then optimize locoregional control and tumor staging. However, for pT1/pT2N0 RC patients, the association between adequate LND and survival remained unclear, and additional therapy was not recommended for these patients with < 12 LND. Thus, we need to evaluate the association between adequate LND and survival in pT1/pT2N0 RC patients.

In the present study, the long-term survival benefit of adequate LND was found in stage I RC patients. Further, subgroup analyses by pT stage suggested that the long-term survival benefit of adequate LND was not in pT1N0 RC patients but in pT2N0 RC patients. The main interpretation for that was the different risk of occult LN metastasis residual between pT1N0 and pT2N0 RC patients (2, 5). A previous study suggested that the incidence of radiographically occult LN metastasis ranges from 6% for low-risk T1 tumors to as high as 65% for poorly differentiated T2 tumors with lymphovascular invasion (LVI) (5). The survival benefit of adequate LND is correlated with the risk of occult LN metastasis residual. The occult LN metastasis residual will increase the risk of locoregional recurrence, which cannot all be amenable to salvage surgical therapy or multimodality therapy (16–18). Previous retrospective studies using the SEER database showed that the survival benefit of radical proctectomy (involved LND) was not found in pT1N0 RC patients but in pT2N0 RC patients referenced to local excision (not involved LND) (9, 12, 19). And the proctectomy can provide better regional control than local excision (LE) for early RC patients, especially for T2 patients (10, 13). Similarly, pT2N0 but not pT1N0 RC patients, will benefit from adequate LND in the present study, which also lent support to the local excision for the treatment of pT1N0 but not for pT2N0 RC patients. Thus, cautions were needed in expanding practice of LE in T2 patients, and adjuvant therapy might be needed for T2 patients treated with LE or proctectomy with inadequate LND. For cT1N0 patients, additional therapy might be needed for those were upstaged to pT2N0 after surgery and with inadequate LND. For cT2N0 patients, adequate LND would be needed.

However, local excision was increasingly used in the treatment of T2N0 RC patients (9, 12). The addition of chemoradiotherapy has been proposed to improve oncologic control (20–25). Previous studies have suggested that T2N0 RC had an equivalent survival between local excision plus adjuvant therapy and radical proctectomy (20–25). A recent study reported that cT2N0 RC treated with neoadjuvant chemoradiotherapy followed by local excision had a comparable survival to those treated with radical proctectomy (22). In the present study, pT2N0 RC patients with adequate LND had statistically significant higher CSS rates and lower CSD rates than those with < 12 LND, which suggested that these patients with < 12 LND were at higher risk, and an additional adjuvant therapy might be needed for these patients to improve oncologic control. However, the actual survival benefit of additional chemoradiotherapy remained unclear for pT2N0 RC patients with < 12 LND in the present study of the limited data. A previous study have indicated a small but statistically significant survival benefit of adjuvant therapy for stage II RC patients, and the benefit of adjuvant therapy is more significant in patients at high-risk, such as in patients with < 12 LND (26, 27). It is reasonable to infer that pT2N0 RC patients with < 12 LND can obtain better local control but a smaller survival benefit after adjuvant therapy referenced to stage II RC patients. Thus, decision-making regarding the use of adjuvant therapy for pT2N0 RC patients with < 12 LND should incorporate patient discussions individualized for the patient and should include the risk of occult nodal metastasis residual and the possible limited benefit and toxicities associated with additional therapy.

The present study with some limitations that should be noticed. Firstly, LVI and tumor budding, which are identified high-risk factors for LN metastasis, were not assessed in the SEER database (10, 11). RC patients with LVI and tumor budding received an inadequately sampled nodes were at higher risk of occult LN metastasis residual, which was associated with worse oncological survival. However, these patients will have a more aggressive LND, and most patients will be excluded in the present study for the positive LN. Previous study suggested that lymph-node distribution rather than number of LN metastasis is a valuable predictor of T1-2 colorectal cancer survival (28). However, the extent of LND was lacked in the SEER database. It was worth further study the prognosis value of the extent of LND, not only the number of LND, in pT1-2N0 RC patients. Secondly, resection margin status which is associated with local recurrence of cancer was also lacking in the database. However, most stage I RC patients with positive resection margin status would choose additional adjuvant therapy if they refused extended surgery, these patients were excluded in the present study. Thirdly, The SEER database also lacked data on the recurrence of cancer and salvage therapy procedures. However, it provides the cause of death to calculate CSS, which is correlated chronologically with cancer recurrence and salvage therapy procedures. Lastly, the nature of the retrospective study. Patient groups were nonrandomized, leading to a selection bias. The imbalance of patients’ demographics and tumor characteristics was found between stage I RC patients with inadequate and adequate LND. Also, significantly different cumulative incidences of competing events were found between stage I RC patients with inadequate and adequate LND in the present study. The inherent selection bias could only be minimally controlled using the multivariable model.

## Conclusions

Despite the limitations and inherent selection bias of retrospective study, this study demonstrated the long-term survival benefit of adequate LND in stage I RC patients. Further subgroup analyses found that the long-term survival benefit of adequate LND was not in pT1N0 but in pT2N0 RC patients. Decision-making regarding the use of adjuvant therapy for pT2N0 RC patients with inadequate LND should balance the possible limited survival benefit against toxicities associated with additional therapy.

## Data availability statement

Publicly available datasets were analyzed in this study. This data can be found here: https://seer.cancer.gov/.

## Ethics statement

Ethical review and approval was not required for the study on human participants in accordance with the local legislation and institutional requirements. Written informed consent for participation was not required for this study in accordance with the national legislation and the institutional requirements.

## Author contributions

LP-L, WD-D, PC-J, and ZL-Z participated in the design of this project, interpretation of data, drafting, and critical revision of the article, and provided final approval of the version to be submitted. LP-L and ZL-Z completed the data collection and analyses. All authors contributed to the article and approved the submitted version.

## Funding

This work was supported in part by Special funding sponsorship of Shandong Province Traditional Chinese Medicine High-level Talent Cultivation Project and Qilu Health Leading Talents Training Project Special Fund.

## Conflict of interest

The authors declare that the research was conducted in the absence of any commercial or financial relationships that could be construed as a potential conflict of interest.

## Publisher’s note

All claims expressed in this article are solely those of the authors and do not necessarily represent those of their affiliated organizations, or those of the publisher, the editors and the reviewers. Any product that may be evaluated in this article, or claim that may be made by its manufacturer, is not guaranteed or endorsed by the publisher.
