# Supplementary material for: Impact of adequate lymph nodes dissection on survival in patients with stage I rectal cancer
Source: Front Oncol. 2022 Nov 18;12:985324. doi: 10.3389/fonc.2022.985324 (PMC9718503; doi:10.3389/fonc.2022.985324)
Supplement: Supplementary file 1 [file DataSheet_1.pdf]

Impact of adequate lymph nodes dissection on survival in patients with stage I rectal cancer

Short running head: Impact of LND on survival
Supplemental Figures: 3 Figures.
Supplemental Tables: 5 Tables.

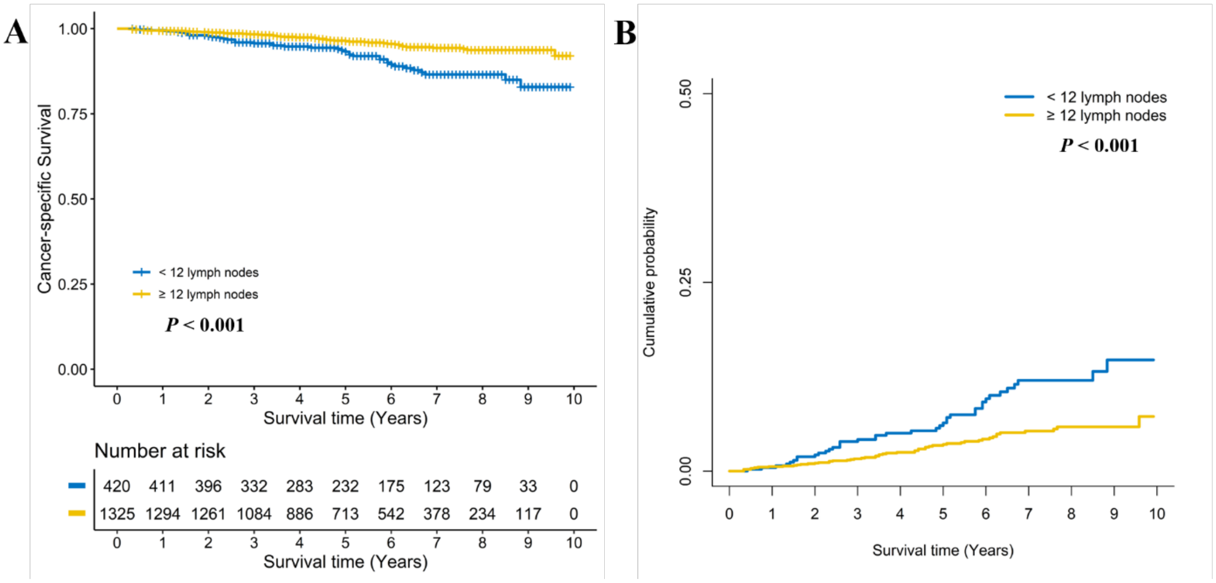

Supplemental Figure 1 Comparison of cancer-specific survival (A) and cumulative probability of cancer-specific death (B) in stage I rectal cancer patients between < 12 and ≥ 12 lymph nodes dissection with exposure starting at > 3 months.

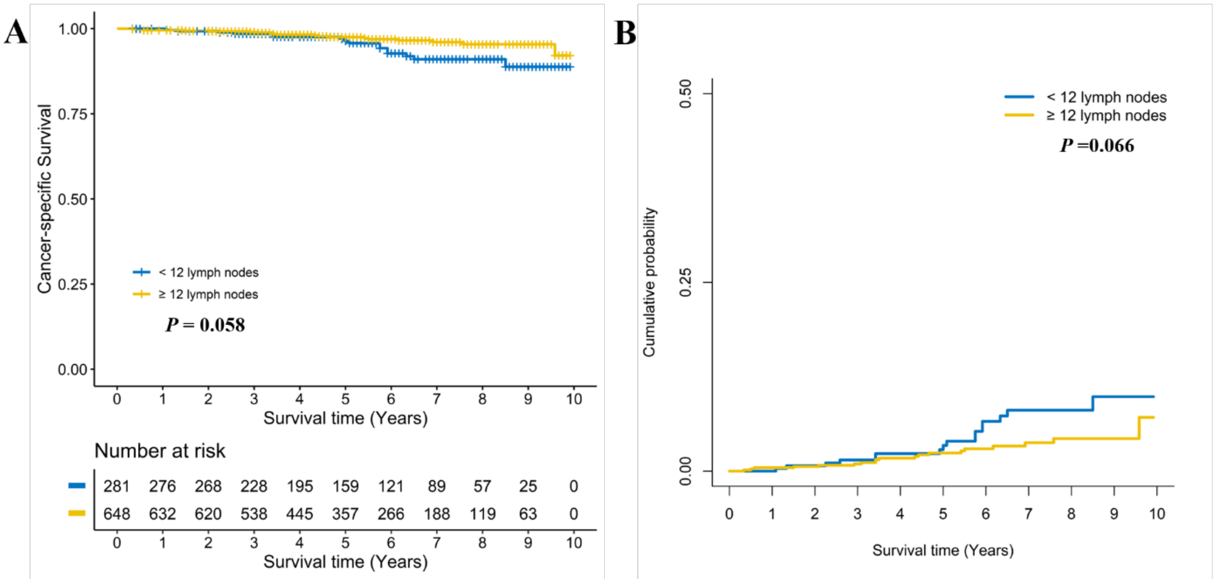

Supplemental Figure 2 Comparison of cancer-specific survival (A) and cumulative probability of cancer-specific death (B) in pT1N0 rectal cancer patients between < 12 and ≥ 12 lymph nodes dissection with exposure starting at > 3 months.

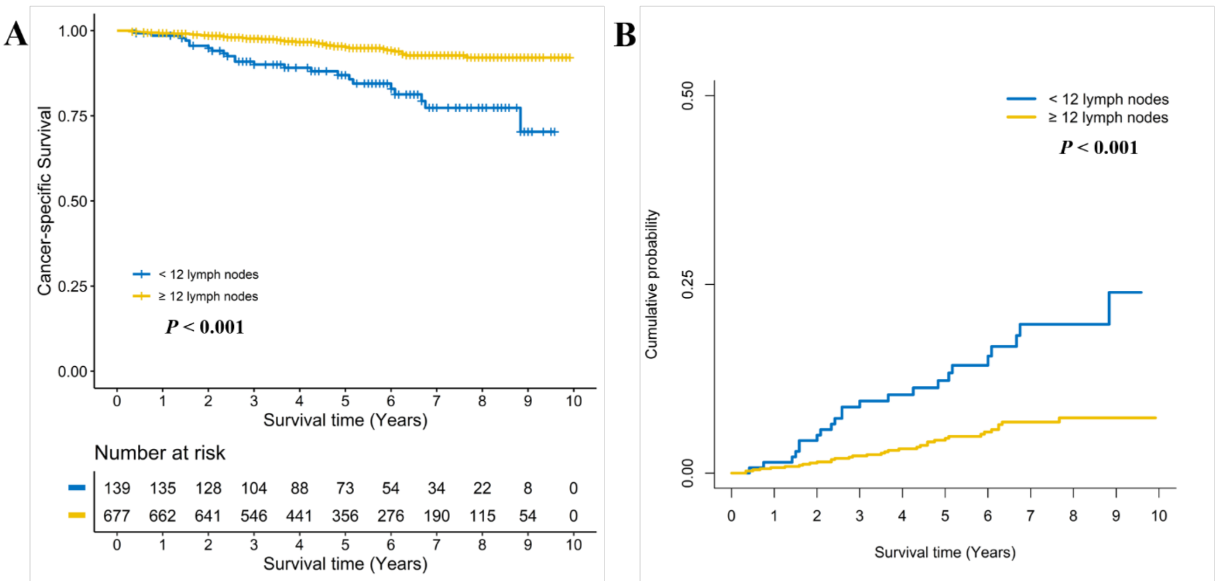

Supplemental Figure 3 Comparison of cancer-specific survival (A) and cumulative probability of cancer-specific death (B) in pT2N0 rectal cancer patients between < 12 and ≥ 12 lymph nodes dissection with exposure starting at > 3 months.

Supplemental Table 1 Clinicopathological differences between pT1N0 rectal adenocarcinoma patients with < 12 and ≥ 12 lymph nodes dissection

Characteristic	Total<br>N = 941	< 12 lymph nodes<br>N = 281	≥ 12 lymph nodes<br>N = 660	*P-value*
Age of diagnosis (years)				0.003
< 50y	107 (11.4)	18 (6.4)	89 (13.5)	
< 60y	340 (36.1)	95 (33.8)	245 (37.1)	
< 70y	288 (30.6)	93 (33.1)	195 (29.5)	
≥ 70y	206 (21.9)	75 (26.7)	131 (19.8)	
Gender				0.266
Female	406 (43.1)	113 (40.2)	293 (44.4)	
Male	535 (56.9)	168 (59.8)	367 (55.6)	
Race				0.425
White	812 (86.3)	246 (87.5)	566 (85.8)	
Black	34 (3.6)	11 (3.9)	23 (3.5)	
Others	95 (10.1)	24 (8.5)	71 (10.8)	
Tumor grade				0.494
Well/ Moderately	793 (84.3)	241 (85.8)	552 (83.6)	
Poor/ Anaplastic	68 (7.2)	16 (5.7)	52 (7.9)	
Unknown	80 (8.5)	24 (8.5)	56 (8.5)	
Tumor size				0.037
≤ 3cm	630 (67.0)	176 (62.6)	454 (68.8)	
> 3 cm	100 (10.6)	27 (9.6)	73 (11.1)	
Unknown	211 (22.4)	78 (27.8)	133 (20.2)	

Characteristic	Total<br>N = 941	< 12 lymph nodes<br>N = 281	≥ 12 lymph nodes<br>N = 660	*P-value*
CEA level				0.997
Negative/ Unknown	879 (93.4)	263 (93.6)	616 (93.3)	
Positive	62 (6.6)	18 (6.4)	44 (6.7)	
Perineural invasion				0.492
Negative/ Unknown	929 (98.7)	279 (99.3)	650 (98.5)	
Positive	12 (1.3)	2 (0.7)	10 (1.5)	

Values are n (%) unless otherwise defined.

Supplemental Table 2 Clinicopathological differences between pT2N0 rectal adenocarcinoma patients with < 12 and ≥ 12 lymph nodes dissection

Characteristic	Total<br>N = 837	< 12 lymph nodes<br>N = 144	≥ 12 lymph nodes<br>N = 693	*P-value*
Age of diagnosis (years)				0.082
< 50y	89 (10.6)	14 (9.7)	75 (10.8)	
< 60y	237 (28.3)	30 (20.8)	207 (29.9)	
< 70y	217 (25.9)	38 (26.4)	179 (25.8)	
≥ 70y	294 (35.1)	62 (43.1)	232 (33.5)	
Gender				0.167
Female	372 (44.4)	56 (38.9)	316 (45.6)	
Male	465 (55.6)	88 (61.1)	377 (54.4)	
Race				0.518
White	726 (86.7)	129 (89.6)	597 (86.1)	
Black	26 (3.1)	3 (2.1)	23 (3.3)	
Others	85 (10.2)	12 (8.3)	73 (10.5)	
Tumor grade				0.292
Well/ Moderately	751 (89.7)	132 (91.7)	619 (89.3)	
Poor/ Anaplastic	74 (8.8)	11 (7.6)	63 (9.1)	
Unknown	12 (1.4)	1 (0.7)	11 (1.6)	
Tumor size				0.010
≤ 3cm	377 (45.0)	79 (54.9)	298 (43.0)	
> 3 cm	442 (52.8)	60 (41.7)	382 (55.1)	
Unknown	18 (2.2)	5 (3.5)	13 (1.9)	

Characteristic	Total<br>N = 837	< 12 lymph nodes<br>N = 144	≥ 12 lymph nodes<br>N = 693	*P-value*
CEA level				0.777
Negative/ Unknown	741 (88.5)	126 (87.5)	615 (88.7)	
Positive	96 (11.5)	18 (12.5)	78 (11.3)	
Perineural invasion				0.839
Negative/ Unknown	813 (97.1)	139 (96.5)	674 (97.3)	
Positive	24 (2.9)	5 (3.5)	19 (2.7)	

Values are n (%) unless otherwise defined.

Supplemental Table 3 The predictors of survival for pT1N0 rectal cancer patients in both multivariate Cox regression model and the multivariate competing risk model.

	Cox regression model		Competing risk model	
Characteristic	adjusted HR (95% CI)	*P-value*	adjusted SHR (95% CI)	*P-value*
Age of diagnosis				
< 50y	Reference		Reference	
< 60y	0.66 (0.20, 2.23)	*0.507*	0.66 (0.18, 2.40)	*0.527*
< 70y	1.29 (0.42, 4.00)	*0.652*	1.28 (0.39, 4.220)	*0.685*
≥ 70y	1.47 (0.47, 4.62)	*0.509*	1.32 (0.40, 4.30)	*0.647*
Gender				
Female	Reference		Reference	
Male	1.28 (0.65, 2.50)	*0.477*	1.26 (0.63, 2.50)	*0.511*
Race				
White	Reference		Reference	
Black	2.17 (0.51, 9.23)	0.293	2.20 (0.55, 8.91)	0.267
Others	1.48 (0.57, 3.85)	0.417	1.54 (0.56, 4.24)	0.401
Tumor grade				
Well/ Moderately	Reference		Reference	
Poor/ Anaplastic	1.18 (0.36, 3.86)	0.789	1.13 (0.34, 3.79)	0.837
Unknown	0.32 (0.04, 2.37)	0.264	0.31 (0.04, 2.41)	0.261
Tumor size				
≤ 3cm	Reference		Reference	

	Cox regression model		Competing risk model	
Characteristic	adjusted HR (95% CI)	*P-value*	adjusted SHR (95% CI)	*P-value*
> 3 cm	1.29 (0.52, 3.19)	0.583	1.28 (0.52, 3.13)	0.593
Unknown	0.69 (0.28, 1.71)	0.423	0.69 (0.27, 1.78)	0.444
CEA level				
Negative/ Unknown	Reference		Reference	
Positive	2.62 (1.13, 6.09)	0.025	2.59 (1.12, 5.96)	0.026
Lymph node dissection				
< 12	Reference		Reference	
≥ 12	0.62 (0.32, 1.19)	0.149	0.63 (0.33, 1.20)	0.158

Supplemental Table 4 The predictors of survival for pT2N0 rectal cancer patients in both multivariate Cox regression model and the multivariate competing risk model.

	Cox regression model		Competing risk model	
Characteristic	adjusted HR (95% CI)	*P-value*	adjusted SHR (95% CI)	*P-value*
Age of diagnosis				
< 50y	Reference		Reference	
< 60y	1.21 (0.32, 4.54)	*0.778*	1.20 (0.32, 4.55)	*0.784*
< 70y	2.22 (0.63, 7.82)	*0.212*	2.24 (0.63, 8.02)	*0.215*
≥ 70y	4.53 (1.37, 14.90)	*0.013*	4.17 (1.26, 13.84)	*0.020*
Gender				
Female	Reference		Reference	
Male	0.82 (0.50, 1.36)	*0.444*	0.81 (0.48, 1.35)	*0.414*
Race				
White	Reference		Reference	
Black	2.81 (0.84, 9.40)	0.093	3.04 (0.96, 9.64)	0.059
Others	0.53 (0.17, 1.71)	0.289	0.54 (0.17, 1.69)	0.290
Tumor grade				
Well/ Moderately	Reference		Reference	
Poor/ Anaplastic	1.53 (0.72, 3.23)	0.267	1.55 (0.73, 3.32)	0.254
Tumor size				
≤ 3cm	Reference		Reference	
> 3 cm	0.81 (0.49, 1.34)	0.407	0.80 (0.49, 1.30)	0.362

	Cox regression model		Competing risk model	
Characteristic	adjusted HR (95% CI)	*P-value*	adjusted SHR (95% CI)	*P-value*
Unknown	0.48 (0.06, 3.56)	0.471	0.43 (0.06, 3.34)	0.419
CEA level				
Negative/ Unknown	Reference		Reference	
Positive	1.56 (0.81, 3.00)	0.188	1.52 (0.79, 2.93)	0.214
Perineural invasion				
Negative/ Unknown	Reference		Reference	
Positive	0.60 (0.08, 4.47)	0.621	0.60 (0.09, 4.26)	0.611
Lymph node dissection				
< 12	Reference		Reference	
≥ 12	0.35 (0.21, 0.58)	< 0.001	0.36 (0.21, 0.62)	< 0.001

Supplemental Table 5 The association between adequate lymph nodes dissection (LND) and survival referenced to inadequate LND in both multivariate Cox regression model and the multivariate competing risk model with exposure starting at > 3 months.

	Cox regression model		Competing risk model	
	adjusted HR (95% CI)	*P-value*	adjusted SHR (95% CI)	*P-value*
All[a]	0.39 (0.25, 0.59)	< 0.001	0.39 (0.25, 0.61)	< 0.001
pT1N0[b]	0.52 (0.27, 1.03)	0.060	0.53 (0.27, 1.02)	0.056
pT2N0[b]	0.31 (0.18, 0.53)	< 0.001	0.31 (0.18, 0.55)	< 0.001

[a] Multivariable analysis adjusted for age of diagnosis, gender, race, pT stage, tumor grade, tumor size, CEA level, and perineural invasion.

[b] Multivariable analysis adjusted for age of diagnosis, gender, race, tumor grade, tumor size, CEA level, and perineural invasion.

[c] Multivariable analysis adjusted for age of diagnosis, gender, race, tumor grade, tumor size, CEA level, and perineural invasion.

HR, hazard ratio; CI, confidence interval; subdistribution HR, SHR.
